# Structural alterations in brainstem, basal ganglia and thalamus associated with parkinsonism in schizophrenia spectrum disorders

**DOI:** 10.1007/s00406-021-01270-y

**Published:** 2021-05-05

**Authors:** Stefan Fritze, Anais Harneit, John L. Waddington, Katharina M. Kubera, Mike M. Schmitgen, Marie-Luise Otte, Lena S. Geiger, Heike Tost, Andreas Meyer-Lindenberg, Robert C. Wolf, Dusan Hirjak

**Affiliations:** 1 Department of Psychiatry and Psychotherapy, Central Institute of Mental Health, Medical Faculty Mannheim, Heidelberg University, Mannheim, Germany; 2 Research Group System Neuroscience in Psychiatry, Department of Psychiatry and Psychotherapy, Central Institute of Mental Health, Medical Faculty Mannheim, Heidelberg University, Mannheim, Germany; 3 School of Pharmacy and Biomolecular Sciences, Royal College of Surgeons in Ireland, Dublin, Ireland; 4 Center for Psychosocial Medicine, Department of General Psychiatry, University of Heidelberg, Heidelberg, Germany

**Keywords:** Sensorimotor domain, Parkinsonism, MRI, Freesurfer, Basal ganglia, Brainstem

## Abstract

The relative roles of brainstem, thalamus and striatum in parkinsonism in schizophrenia spectrum disorder (SSD) patients are largely unknown. To determine whether topographical alterations of the brainstem, thalamus and striatum contribute to parkinsonism in SSD patients, we conducted structural magnetic resonance imaging (MRI) of SSD patients with (SSD-P, *n* = 35) and without (SSD-nonP, *n* = 64) parkinsonism, as defined by a Simpson and Angus Scale (SAS) total score of ≥ 4 and < 4, respectively, in comparison with healthy controls (*n* = 20). FreeSurfer v6.0 was used for segmentation of four brainstem regions (medulla oblongata, pons, superior cerebellar peduncle and midbrain), caudate nucleus, putamen and thalamus. Patients with parkinsonism had significantly smaller medulla oblongata (*p* = 0.01, false discovery rate (FDR)-corrected) and putamen (*p* = 0.02, FDR-corrected) volumes when compared to patients without parkinsonism. Across the entire patient sample (*n* = 99), significant negative correlations were identified between (a) medulla oblongata volumes and both SAS total (*p* = 0.034) and glabella-salivation (*p* = 0.007) scores, and (b) thalamic volumes and both SAS total (*p* = 0.033) and glabella-salivation (*p* = 0.007) scores. These results indicate that brainstem and thalamic structures as well as basal ganglia-based motor circuits play a crucial role in the pathogenesis of parkinsonism in SSD.

## Introduction

Parkinsonism in schizophrenia spectrum disorders (SSD) is a multidimensional syndrome characterized by tremor, rigor, akinesia and hypersalivation [1–3]. The neurobiological mechanisms underlying parkinsonism in SSD are thought to reflect an interplay between spontaneous (i.e. disease-related) and antipsychotic drug-exacerbated movement disorder [1, 4–8]. Previous multimodal magnetic resonance imaging (MRI) and other studies have considered several putative neurobiological mechanisms including prominent striatal contributions [9] and disturbed structural–functional coupling between cortical and subcortical systems, particularly in cortical-striatal-thalamocortical networks [5, 6, 10, 11]. However, previous structural MRI studies used techniques that were unable to account sufficiently for the convoluted morphological relationships among brainstem, striatal and thalamic structures [12]. In addition, there is a paucity of evidence concerning structural brainstem abnormalities in SSD patients with parkinsonism.

Therefore, the present MRI study used both a categorical and a dimensional (correlational) approach to investigate the relationships between morphological variations of subcortical structures [medulla oblongata, pons, superior cerebellar peduncle (SCP), midbrain, caudate nucleus, putamen and thalamus] and parkinsonism assessed with the Simpson and Angus Scale (SAS) [26] in SSD patients. Currently, the SAS is the only instrument that allows robust estimation of parkinsonism in SSD patients. The SAS estimates rigor, tremor, hypokinesia, hypersalivation, and glabellar tap. Particularly noteworthy is glabella tap, as this is ascribed to frontal release signs [13] and is considered an intrinsic sensorimotor sign (i.e. reflecting vulnerability to and emergence of illness) that is not related to effects of medication. This study had two main hypotheses: first, using a categorical approach, we hypothesized that brainstem structures, caudate nucleus, putamen and thalamus volumes will differ between SSD patients with (SSD-P, SAS total score ≥ 4) and without (SSD-nonP, SAS total score < 4) parkinsonism. Second, using a dimensional approach (i.e. across increasing severities of parkinsonism) and in accordance with a model of dopaminergic-driven subcortical-cortical motor circuitry [14–17], we hypothesized that the volumes of these structures will be associated with distinct symptom dimensions of parkinsonism.

## Methods

### Study participants

We evaluated a total of 111 right-handed [18] subjects satisfying DSM-IV-TR [19] criteria for schizophrenia (*n* = 104) or schizoaffective disorder (*n* = 7) [20, 21]. Diagnoses were made by staff psychiatrists and confirmed using the German versions of the Structured Clinical Interview for DSM-IV-TR axis I and II disorders (SCID) and examination of the case notes (D.H. and S.F.). Patients were excluded if: (1) they were aged < 18 or > 65 years; (2) they had a history of brain trauma or neurological disease (especially movement disorders); or (3) they had shown alcohol/substance use disorder within 12 months prior to participation.

Twenty-eight healthy right-handed control subjects (HC) were also studied. Exclusion criteria included MRI contraindications, a history of psychiatric, neurological, cardiovascular or metabolic illness, prior head trauma, and current alcohol or drug abuse. None of the control subjects had a first-degree relative with a psychiatric disorder or were receiving psychopharmacological treatment.

The study protocol adhered to the Declaration of Helsinki. The local Research Ethics Committee (Medical Faculty at Heidelberg University, Germany) approved the study. We obtained written informed consent from all study participants after all aims and procedures of the study had been fully explained.

### Clinical assessment

Patients were recruited and examined by SF and DH within 1 week after partial remission of psychotic symptoms. The duration between the evaluation of psychopathology (Positive and Negative Syndrome Scale [PANSS] [22], Brief Psychiatric Rating Scale [BPRS] [23], Clinical Global Impression Scale [CGI] [24]), functional capacity (Global assessment of functioning [GAF] [25]), sensorimotor assessment (Simpson-Angus Scale (SAS) [26] and Northoff Catatonia Rating Scale (NCRS) [27, 28]) and MRI examination was less than 3 days. At the time of examination, none of the SSD patients were treated with benzodiazepine or anticholinergic medication and all but 4 patients were receiving stable antipsychotic medication for at least 2 weeks. Daily doses of antipsychotic medication were converted to olanzapine equivalents (OLZ) [29].

For assessment of parkinsonism, we used the SAS [26]. We then excluded 12 SSD patients from the original study sample (111 − 12 = 99) to create two well-balanced (in terms of age, sex, education and OLZ-equivalent dose) groups of SSD patients with parkinsonism (SSD-P; SAS total score ≥ 4, *n* = 35) and without parkinsonism (SSD-nonP; SAS total score < 4, *n* = 64) according to previously described cut‐off criteria [30]. The patient groups were carefully matched with respect to sex and education because both variables can influence sensorimotor functioning in SSD [2, 31]. Similarly, we excluded 8 HC from the original sample (28 − 8 = 20) to create a well-matched (in terms of age, sex and education) control group (*n* = 20). Finally, we followed a correlative approach, assuming dimensional symptom expression along a neurobiological continuum in SSD patients with various degrees of parkinsonism (*n* = 99) [32].

### MRI data acquisition

MRI scans were acquired at the Central Institute of Mental Health, Mannheim, Germany, using a 3.0 T Siemens Trio whole-body imaging system and a T1-weigthed magnetization-prepared rapid gradient-echo (MP-RAGE) sequence with the following parameters: repetition time (ms): 2530; echo time (ms): 3.8; inversion time (ms): 1100; flip angle: 7°; number of averages: 1; slice thickness (mm): 1; image columns: 256; image rows: 256; phase encoding direction: ROW; voxel size *x* (mm): 1; voxel size *y* (mm): 1; number of volumes: 1; number of slices: 176; number of files: 176.

### Image processing

FreeSurfer v6.0 [33] was used for the segmentation of four brainstem regions and the caudate nucleus, putamen and thalamus [34–36]; for further details on these methods see (http://surfer.nmr.mgh.harvard.edu/). This segmentation tool is able to perform volumetric segmentation of four brainstem regions (medulla oblongata, pons, SCP and midbrain) from T1 (MP-RAGE) images using a Bayesian algorithm that relies on a probabilistic atlas of the brainstem and neighboring brain structures [12]. Furthermore, this tool uses soft segmentation, i.e. a voxel can be assigned to multiple structures/tissues, which results in improved performance regarding partial volume effects from surrounding cerebrospinal fluid [12]. The volumes of the caudate nucleus, putamen and thalamus were performed using the aseg.stats command. Since we did not have an explicit laterality hypothesis, we calculated a mean value from the left and right volumes of these three structures. Finally, the estimated total intracranial volume (eTIV) was calculated with FreeSurfer as recommended. FreeSurfer exploits a relationship between intracranial volume and linear transformation to MNI305 space (talairach.xfm) as described previously [37].

### Statistical analysis

We used SPSS version 26. Initially, a descriptive analysis for demographic, clinical and volumetric data in SSD-P and SSD-nonP patients (Table 1) was performed. Then, homogeneity of variance for each subcortical region and SAS scores in both patient groups was evaluated and confirmed using Levene’s test.

**Table 1:** Demographic, clinical and sensorimotor characteristics for SSD patients with (SSD-P) and without (SSD-nonP) parkinsonism and healthy controls (HC)

Variable	SSD-P (*n* = 35)	SSD-nonP (*n* = 64)	HC (*n* = 20)	*F/χ*^*2*^*/t*	*df*	*p*
Age (years)^a^	40.9 ± 11.2	40.7 ± 9.7	40.7 ± 13.6	0.002	2116	0.99
Sex (male/female)^b^	19/16	32/32	9/11	0.44	2116	0.79
Education (years)^a^	13.2 ± 2.9	13.2 ± 2.6	13.5 ± 1.8	0.06	2116	0.93
Duration of illness (years)^c^	13.7 ± 12.4	10.6 ± 10.4	–	1.30	97	0.19
OLZ^a^	20.1 ± 11.3	17.2 ± 8.9	0.0 ± 0.0	1.69	97	0.17
Duration of illness (years)^c^	13.7 ± 12.4	10.6 ± 10.4	–	1.3	97	0.19
PANSS-P score^c^	13.4 ± 5.6	16.3 ± 7.6	–	1.96	97	0.051
PANSS-N score^c^	17.3 ± 7.8	15.27 ± 7.1	–	1.34	97	0.18
PANSS-G score^c^	32.3 ± 8.4	35.2 ± 11.4	–	1.39	97	0.19
PANSS-total score^c^	62.9 ± 17.0	66.7 ± 22.0	–	0.88	97	0.37
GAF score^c^	68.6 ± 16.5	71.2 ± 16.9	–	0.75	97	0.45
CGI-S^c^	3.9 ± 1.0	3.9 ± 0.9	–	0.16	97	0.86
*SAS hypokinesia*^c^	1.1 ± 0.7	0.4 ± 0.5	–	5.72	97	**< 0.0001**
*SAS rigidity*^c^	2.6 ± 2.2	0.3 ± 0.5	–	8.38	97	**< 0.0001**
*SAS tremor*^c^	0.9 ± 0.7	0.4 ± 0.5	–	4.49	97	**< 0.0001**
*SAS glabella-salivation*^c^	1.3 ± 0.9	0.5 ± 0.7	–	4.92	97	**< 0.0001**
*SAS total score*^c^	5.9 ± 2.2	1.5 ± 1.1	–	13.43	97	**< 0.0001**
*NCRS motor*^c^	1.1 ± 1.5	0.6 ± 0.8	–	2.30	97	**0.024**
*NCRS affective*^c^	1.8 ± 1.4	1.5 ± 1.9	–	1.0	97	0.31
*NCRS behavioral*^c^	1.2 ± 1.5	0.7 ± 1.1	–	1.88	97	0.062
*NCRS total score*^c^	4.1 ± 3.4	2.7 ± 3.0	–	2.16	97	**0.03**
*eTIV*	1.47 ± 0.20 × 10^6^	1.50 ± 0.20 × 10^6^	1.51 ± 0.22 × 10^6^	0.29	2116	0.74

Data are mean ± standard deviation. Significant results (*p* < 0.05) are displayed in bold font

*SSD* schizophrenia spectrum disorders, *SSD-P* SSD patients with parkinsonism, *SSD-nonP* SSD patients without parkinsonism, *HC* healthy controls, *OLZ* mean daily dose of antipsychotics in olanzapine equivalents, *PANSS* Positive and Negative Syndrome Scale, as -total score, and -positive (P), -negative (N) and -general (G) subscale scores, *BPRS* Brief Psychiatric Rating Scale, *GAF* Global Assessment of Functioning, *CGI-S* Clinical Global Impression Scale for Schizophrenia, *SAS* Simpson and Angus Scale, with total and subscale scores, *NCRS* Northoff Catatonia Rating Scale, with total and subscale scores, *eTIV* estimated total intracranial volume (mm^3^)

^a^F, df,and *p* values were obtained using ANOVA

^b^*χ*^*2*^*,* df and *p* values were obtained using the Chi-squared test

^c^t, df and *p* values were obtained using independent sample *t* tests (two-tailed)

Group differences: in a first step, one-way analysis of variance (ANOVA), based on the General Linear Model procedure as implemented in SPSS, was used to identify any significant differences between the means of the three study groups. In a second step, we performed a one-way ANCOVA using eTIV, OLZ and PANSS-P scores as covariates to identify any significant differences between SSD-P and SSD-nonP patients. Then, we performed a one-way ANCOVA using eTIV and OLZ as covariates to determine whether there are any significant differences between SSD-P and SSD-nonP patients and HC.

Structure-symptom associations: in a third step, partial correlations (Pearson coefficient, two-tailed) using age, sex, OLZ, eTIV, and PANSS-N scores as covariates were run to determine the relationships between medulla oblongata and putamen volumes and SAS scores in the whole sample of SSD patients (*n* = 99). A nominal significance threshold of *p* ≤ 0.05 was defined. Finally, out of concern that some parkinsonian features might be misinterpreted as catatonic symptoms, thus inflating SAS scores, in a further step NCRS total scores were included as covariates in all structure-symptom analyses. To account for false-positive findings within identified between-group differences and structure-symptom associations, *p*-values were adjusted after each step using the false discovery rate (FDR; *p* ≤ 0.05) correction [38].

## Results

### Clinical, demographic and volumetric characteristics

Demographic and clinical characteristics of the three study groups are shown in Table 1. Of the 99 SSD patients analyzed, 35 (35.4%) were operationally defined as having parkinsonism (SSD-P, SAS total score ≥ 4) and compared with 64 (64.6%) who were operationally defined as not having parkinsonism (SSD-nonP, SAS total score < 4); SSD-P and SSD-nonP patients were well balanced (propensity matched) for age, sex, education and OLZ. In further between-group analyses, these 35 SSD-P patients and 64 SSD-nonP patients were each compared with the 20 HC that were similarly well matched for age, sex and education.

### Group differences

**Fig. 1:**
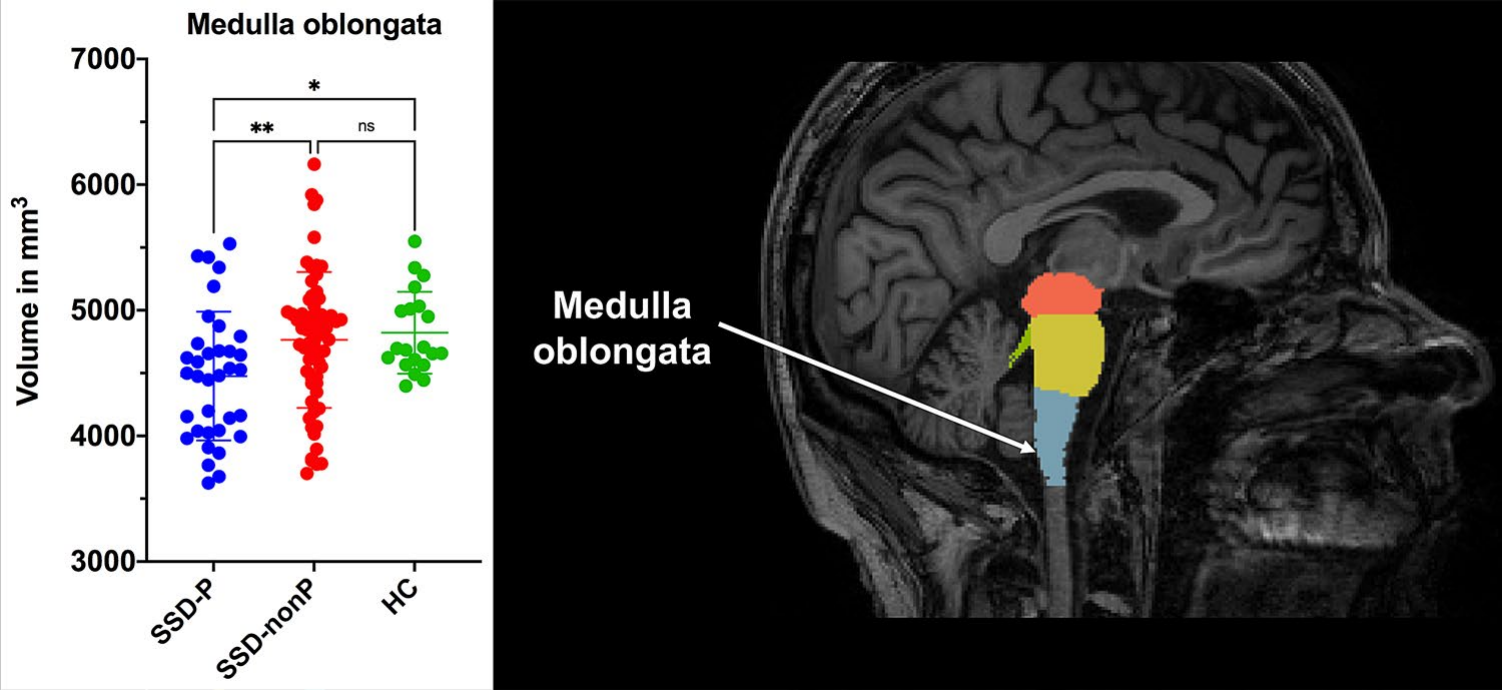
Scatter plot showing medulla oblongata volumes in SSD patients with (SSD-P, *n* = 35) and without (SSD-nonP, *n* = 64) parkinsonism and healthy controls (HC, *n* = 20). Significant between-group differences are designated with one asterisk (*p* < 0.05) or two asterisks (*p* < 0.01); *ns* not significant

**Fig. 2:**
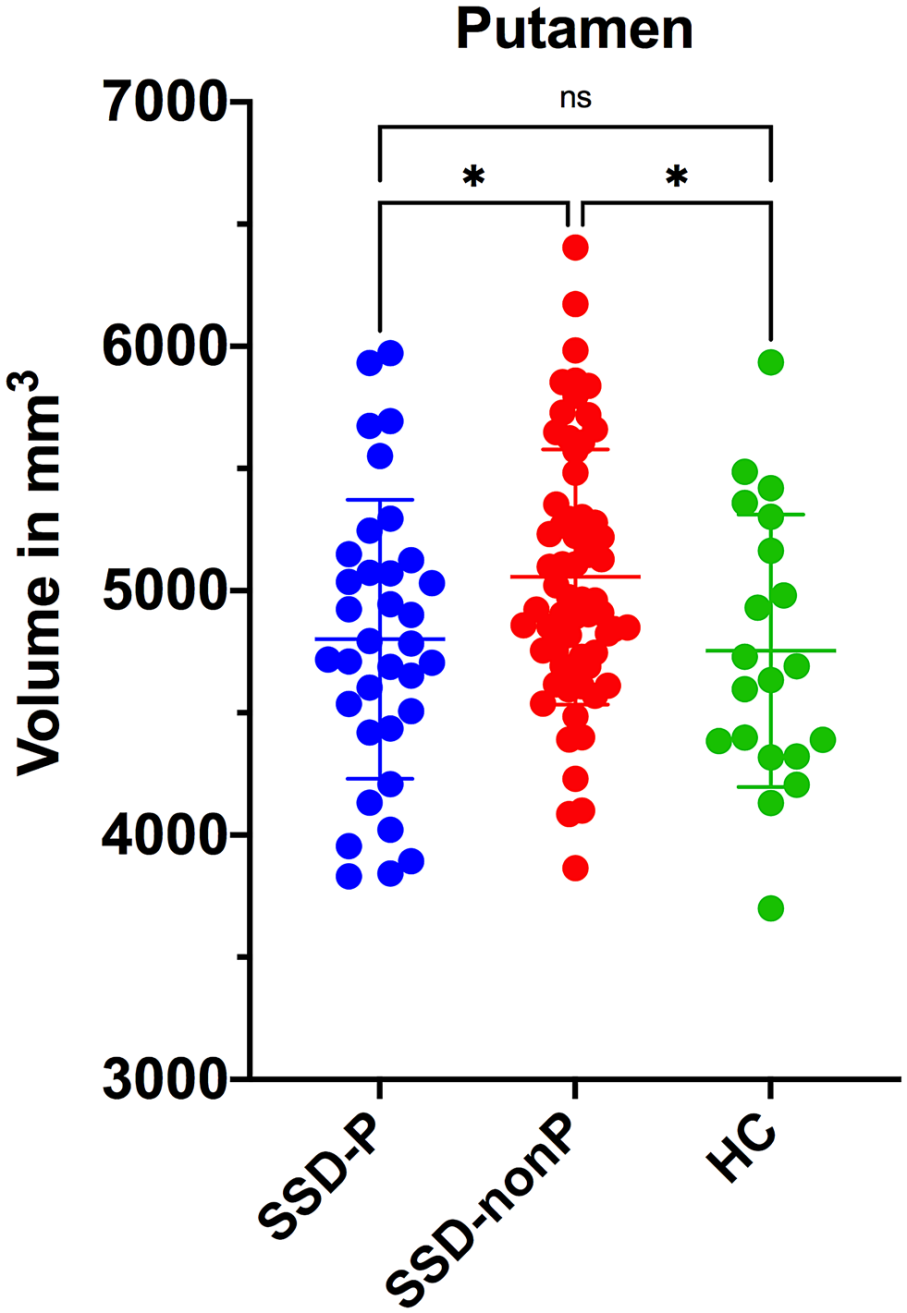
Scatter plot showing putamen mean volumes in SSD patients with (SSD-P, *n* = 35) and without (SSD-nonP, *n* = 64) parkinsonism and healthy controls (HC, *n* = 20). Significant between-group differences are designated with one asterisk (*p* < 0.05); *ns* not significant

**Fig. 3:**
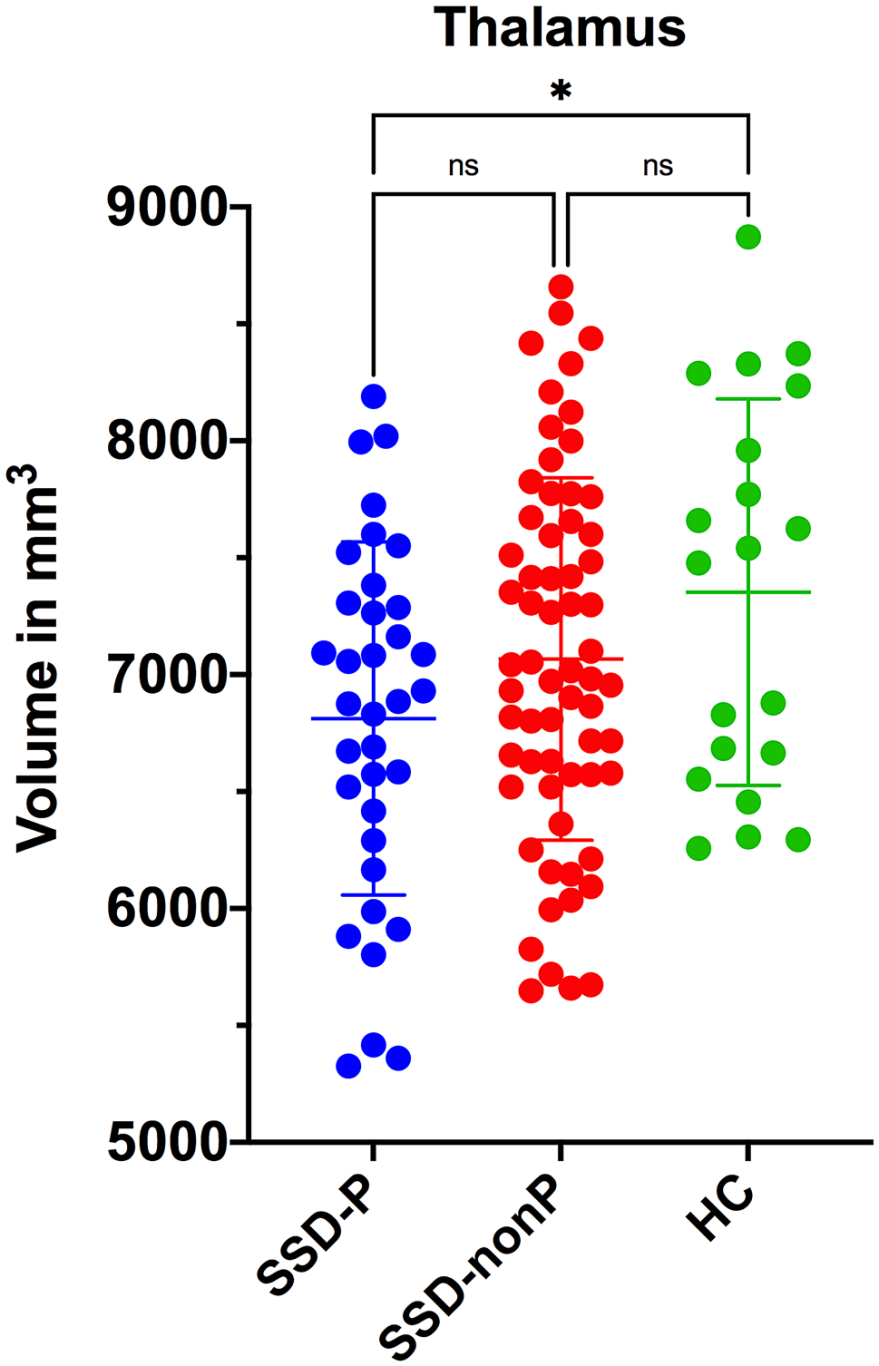
Scatter plot showing thalamus mean volumes in SSD patients with (SSD-P, *n* = 35) and without (SSD-nonP, *n* = 64) parkinsonism and healthy controls (HC, *n* = 20). Significant between-group differences are designated with one asterisk (*p* < 0.05); *ns* not significant

First, on ANOVA there were significant overall differences between the three study groups in the medulla oblongata (*F*_(2,116)_ = 4.53, *p* = 0.01), putamen (*F*_(2,116)_ = 3.14, *p* = 0.04) and thalamic (*F*_(2,116)_ = 3.77, *p* = 0.02) volumes (Table 2). There were no significant overall group differences in the midbrain, SCP, pons, whole brainstem or caudate nucleus (*p* > 0.05) volumes. Least significant difference (LSD) post hoc tests were then applied for individual group comparisons. In medulla oblongata (Fig. 1), volume in SSD-nonP patients did not differ from HC, while volume in SSD-P patients was decreased relative to HC (*p* = 0.01); volume in SSD-P patients was reduced relative to SSD-nonP patients (*p* = 0.007). In putamen (Fig. 2), volume in SSD-nonP patients was increased relative to HC (*p* = 0.03), while volume in SSD-P patients did not differ from HC; volume in SSD-P patients was decreased relative to SSD-nonP patients (*p* = 0.02). In thalamus (Fig. 3), volume in SSD-P patients was decreased relative to HC (*p* = 0.01), while volume in SSD-nonP was intermediate and differed from neither SSD-P nor HC.

**Table 2:** Brainstem and basal ganglia structural volumes in SSD patients with (SSD-P) and without (SSD-nonP) parkinsonism and healthy controls (HC)

Structure	SSD-P	SSD-nonP	HC	*p* values for ANOVA and LSD tests	p values for ANCOVA		
	(*n* = 35)	(*n* = 64)	(*n* = 20)		SSD-P vs. SSD-nonP^a^	SSD-P vs. HC^b^	SSD-nonP vs. HC^b^
Medulla oblongata	4476 ± 512	4764 ± 541	4821 ± 325	ANOVA: **0.01** LSD: SSD-P < SSD-nonP **0.007**; SSD-P < HC **0.01**; SSD-nonP vs. HC 0.65	0.01*	**0.04**	0.61
Pons	14,329 ± 1888	14,897 ± 1752	15,150 ± 1320	0.17	–	**–**	–
SCP	261 ± 54	277 ± 59	295 ± 52	0.08	–	–	–
Midbrain	5762 ± 558	6006 ± 508	6003 ± 509	0.07	**–**	–	**–**
Whole brainstem	24,829 ± 2792	25,945 ± 2662	26,270 ± 2028	0.07	–	**–**	–
Caudate^c^	3432 ± 437	3638 ± 465	3541 ± 458	0.10	**–**	–	–
Putamen^c^	4802 ± 571	5056 ± 522	4754 ± 558	ANOVA: **0.02** LSD: SSD-P < SSD-nonP **0.02**; SSD-P vs. HC 0.75; SSD-nonP > HC **0.03**	0.02*	0.94	0.72
Thalamus^c^	6813 ± 756	7066 ± 775	7353 ± 827	ANOVA: **0.04** LSD: SSD-P vs. SSD-nonP 0.12; SSD-P < HC **0.01**; SSD-nonP vs. HC 0.15	0.14	**0.02**	0.58

Data are mean ± standard deviation (mm^3^). Significant differences (*p* < 0.05) in means between all three groups using one-way ANOVA are indicated in bold. Significant differences (*p* < 0.05) in means between two groups using ANCOVA are indicated in bold

*LSD* Fisher’s least significant difference test, *SCP* superior cerebellar peduncle, *eTIV* estimated total intracranial volume

^a^*F* and *p* values are for ANCOVA with OLZ, eTIV and PANSS-P score as covariates (see Table 1)

^b^*F* and *p* values are for ANCOVA with OLZ and eTIV as covariates (see Table 1). ANCOVA were followed by Benjamini & Hochberg correction for false discovery rate [38] to test the differences among groups. Values surviving Benjamini & Hochberg correction are indicated by an asterisk (*)

^**c**^Mean of bilateral values

Second, on ANCOVA (Table 2), there were significant differences (1) between SSD-P and SSD-nonP patients in medulla oblongata (*p* = 0.01) and putamen (*p* = 0.02) volumes, and (2) between SSD-P patients and HC in medulla oblongata (*p* = 0.04) and thalamic (*p* = 0.02) volumes, while these volumes did not differ between SSD-nonP patients and HC.

### Structure-symptom associations

Higher SAS total and SAS glabella-salivation scores were each negatively associated with medulla oblongata volume (*r* = − 0.219, *p* = 0.034 and *r* = − 0.277, *p* = 0.007, respectively) and thalamic volume (*r* = − 0.220, *p* = 0.033 and *r* = − 0.274, *p* = 0.007, respectively); only the associations between SAS glabella-salivation scores and medulla oblongata and thalamic volumes survived FDR correction (*p* < 0.05). Finally, using NCRS total scores as a covariate, higher SAS total scores were negatively associated with medulla oblongata volumes (*r* = − 0.212, *p* = 0.041) and SAS glabella-salivation scores were negatively associated with medulla oblongata volumes (*r* = − 0.267, *p* = 0.009) and thalamic volumes (*r* = − 0.259, *p* = 0.012); only the association between SAS glabella-salivation scores and medulla oblongata and thalamic volumes survived FDR correction (*p* ≤ 0.05).

## Discussion

Using subcortical segmentation tools implemented in FreeSurfer v6.0, we investigated structural differences in brainstem structures, caudate nucleus, putamen and thalamus between SSD patients with and without parkinsonism in comparison with healthy controls. Three main findings emerged: firstly, SSD-P patients showed a reduced volume of the medulla oblongata and putamen compared to SSD-nonP and HC; secondly, SSD-P patients did not show the increase in volume of the putamen that was evident in SSD-nonP patients compared to HC; thirdly, SAS glabella-salivation scores were associated negatively with medulla oblongata and thalamic volumes in the whole SSD sample.

### Group differences

To our knowledge, this is the first MRI study that specifically aimed to compare volumes of subcortical sensorimotor brain regions in SSD patients with and without parkinsonism and HC. In line with our hypothesis, SSD-P patients showed reduced volumes of the medulla oblongata and putamen compared to SSD-nonP patients. These findings are important for better understanding the pathogenesis of parkinsonism for several reasons:

First, the medulla oblongata is the lowest part of the brainstem and contains multiple nuclei (e.g. nucleus ambiguus, the dorsal vagal motor nucleus and the raphe nucleus) and tracts that connect the spinal cord with the forebrain. In particular, the medulla oblongata contains the inferior olivary nuclei, the pyramidal decussation of the motor pathways, and the spinothalamic tract [39]. The inferior olivary nuclei are responsible for proprioception, muscle tension and motor intention [39]. These nuclei are also closely connected to the cerebellum. The medulla oblongata is a location where the majority of motor pathways from the cortex decussate to form the corticospinal tract (CST). In addition to the CST, the medulla oblongata includes the spinothalamic tract and serves as a switch point between motor pathways from the cortex, thalamus, cerebellum, and spinal cord. In line with this functionality, atrophy of the brainstem and medulla oblongata [40] are associated with neurological disorders characterized by parkinsonian symptoms [41]. More recently, Fritze and colleagues [42] found that medulla oblongata volumes are associated significantly with motor coordination abilities in SSD. Taken together, structural abnormalities of the medulla oblongata can result in aberrant signal transmission between sensorimotor regions that lead to the development of sensorimotor abnormalities, and the present findings extend this to parkinsonism in SSD. Patients with SSD show an overall decrease in volume of the medulla oblongata, which is subject to broad genetic regulation [43] in a manner that may differ between SSD-P and SSD-nonP patients.

Second, SSD-P patients did not show the increase in putamen volume that was evident in SSD-nonP patients relative to HC. The putamen, together with the caudate nucleus, is now considered to play a critical role in the pathobiology of SSD [44] in addition to its classical role in sensorimotor abnormalities; it is interconnected with the primary motor cortex and the supplementary motor area and hence has a fundamental role in motor control [45]. Previous reports have indicated that overall increases in the volume of the putamen in SSD patients involve a trophic effect of treatment with antipsychotic drugs at a site rich in D2 dopamine receptors, which are the primary targets for antipsychotics [46–49]. Therefore, such a trophic effect of antipsychotic treatment may contribute to the increase in putamen volume found in SSD-nonP patients, perhaps as a compensatory mechanism to overcome impaired sensorimotor functioning intrinsic to the disease process of SSD. In contrast, the absence of an increase in putamen volume in SSD-P patients may reflect a reduced capacity to invoke such a response to antipsychotic treatment, which is reflected in the overt sensorimotor dysfunction of parkinsonism. Increase in putamen volume appears to be under specific genetic regulation in a manner that is weakened in SSD [50] and may differ between SSD-P and SSD-nonP patients.

Third, SSD patients showed a graded decrease in thalamic volume (SSD-P < SSD-nonP < HC). The thalamus is an important component in cortical-striatal-thalamocortical networks that have fundamental roles in the sensorimotor function and movement disorder [10] and the volumes of several thalamic nuclei are known to be decreased in SSD [51]. Decrease in thalamic volume is associated with polygenic risk for SSD [52], which may vary between SSD-P and SSD-nonP patients.

### Structure-symptom relationships

The SAS glabella-salivation scores were negatively associated with medulla oblongata and thalamic volumes. Interestingly, glabellar tap is a frontal release sign, which can be detected early after birth but disappears in the process of further brain development [13]. Consequently, the origin of the glabellar tap sign, like other frontal release signs associated with movement disorder in SSD [53], may be ascribed not to antipsychotic treatment effects but, rather, to frontal lobe dysfunction intrinsic to the underlying disease process [54]. In the present study, the negative association between glabellar tap scores and medulla oblongata and thalamic volumes suggests a disturbance of bottom-up modulation via subcortical-extrapyramidal circuits [55–57] leading to disinhibition of cortical sensorimotor regions (as reflected by frontal release signs), particularly on taking into account thalamic function as a ‘gatekeeper’ [58].

Salivation is under M_3_- and M_4_-mediated cholinergic control and the antipsychotic clozapine is associated with hypersalivation, while other antipsychotic drugs are more prone to anticholinergic side effects such as dry mouth [59]. Hypersalivation in SSD-P can be due to increased production or decreased swallowing, the latter possibly due to bradykinesia or clozapine-associated decrease in laryngeal peristalsis [59]. However, the influence of clozapine on the salivation item of the SAS in our sample seems rather limited when taking into account that only 19 of 99 patients (19%) received clozapine treatment.

## Limitations

Despite the advantages of the study (sample size, systematic comparisons between HC, SSD-nonP and SSD-P patients), there are some limitations: first, the cross-sectional design does not allow conclusions about the stability or dynamics of the findings over time, as both parkinsonian symptoms and subcortical structure and function may vary over the course of illness. Second, our study included SSD patients receiving antipsychotic medication. Although antipsychotic drugs might still be considered as potentially influencing sensorimotor assessment, the contribution of spontaneous sensorimotor abnormalities intrinsic to the disease process of SSD [60] and the effects of such treatment to exacerbate such intrinsic abnormalities (rather than ‘cause’ them de novo) are increasingly recognized [1, 3, 61]. Thus, though it might be argued that there is no way to reliably differentiate spontaneous and drug-induced parkinsonian symptoms in patients receiving antipsychotic medication, this appears to be a false dichotomy given that the latter appear to be an antipsychotic-induced exacerbation of the former within unitary network dysfunction [3, 5, 7]. Furthermore, SSD and parkinsonian movement disorder share genetic risk factors and thus appear to involve overlapping pathobiologies [62]. To clarify these issues would require longitudinal instrumental and momentary ecological assessments in both antipsychotic-naïve and treated SSD patients, including periods both on- and off-medication.

## Conclusion

These relationships between parkinsonism in SSD and volumes of the medulla oblongata, putamen and thalamus should not be considered independently. As the medulla oblongata enjoys functionally important efferent and afferent connectivity with the thalamus and putamen as well as the cortex, it interacts closely across several components in the cortical-striatal-thalamocortical networks that have been implicated in the pathobiology of parkinsonian movement disorder [10, 63, 64]. These three brain structures involved in dopaminergically based motor circuits appear to play an important, integrative role in the pathobiology of parkinsonism in SSD.

## Data Availability

All original data are on record and accessible to inspection, but are not available publicly based on local and national data protection regulations.

## Code availability

All software used in the analyses is based on publicly available code.

## References

[1] Pappa S, Dazzan P (2009). Spontaneous movement disorders in antipsychotic-naive patients with first-episode psychoses: a systematic review. Psychol Med.

[2] Peralta V, Basterra V, Campos MS, de Jalon EG, Moreno-Izco L, Cuesta MJ (2012). Characterization of spontaneous parkinsonism in drug-naive patients with nonaffective psychotic disorders. Eur Arch Psychiatry Clin Neurosci.

[3] Walther S, Strik W (2012). Motor symptoms and schizophrenia. Neuropsychobiology.

[4] Hirjak D, Thomann PA, Kubera KM, Wolf ND, Sambataro F, Wolf RC (2015). Motor dysfunction within the schizophrenia-spectrum: a dimensional step towards an underappreciated domain. Schizophr Res.

[5] Whitty PF, Owoeye O, Waddington JL (2009). Neurological signs and involuntary movements in schizophrenia: intrinsic to and informative on systems pathobiology. Schizophr Bull.

[6] Wolf RC, Rashidi M, Fritze S, Kubera KM, Northoff G, Sambataro F, Calhoun VD, Geiger LS, Tost H, Hirjak D (2020). A neural signature of parkinsonism in patients with schizophrenia spectrum disorders: a multimodal mri study using parallel ica. Schizophr Bull.

[7] Waddington JL (2020). Psychosis in parkinson's disease and parkinsonism in antipsychotic-naive schizophrenia spectrum psychosis: clinical, nosological and pathobiological challenges. Acta Pharmacol Sin.

[8] Wolf RC, Kubera KM, Waddington JL, Schmitgen MM, Fritze S, Rashidi M, Thieme CE, Sambataro F, Geiger LS, Tost H, Hirjak D (2021). A neurodevelopmental signature of parkinsonism in schizophrenia. Schizophr Res.

[9] Northoff G (2002). What catatonia can tell us about "top-down modulation": A neuropsychiatric hypothesis. Behav Brain Sci.

[10] McGregor MM, Nelson AB (2019). Circuit mechanisms of parkinson's disease. Neuron.

[11] Wolf RC, Kubera KM, Waddington JL, Schmitgen MM, Fritze S, Rashidi M, Thieme CE, Sambataro F, Geiger LS, Tost H, Hirjak D (2021). A neurodevelopmental signature of parkinsonism in schizophrenia. Schizophr Res.

[12] Iglesias JE, Van Leemput K, Bhatt P, Casillas C, Dutt S, Schuff N, Truran-Sacrey D, Boxer A, Fischl B, Alzheimer's Disease Neuroimaging I (2015). Bayesian segmentation of brainstem structures in mri. Neuroimage.

[13] Hyde TM, Goldberg TE, Egan MF, Lener MC, Weinberger DR (2007). Frontal release signs and cognition in people with schizophrenia, their siblings and healthy controls. Br J Psychiatry.

[14] Hirjak D, Kubera KM, Wolf RC, Northoff G (2020). Going back to kahlbaum's psychomotor (and gabaergic) origins: Is catatonia more than just a motor and dopaminergic syndrome?. Schizophr Bull.

[15] Wei W, Wang XJ (2016). Inhibitory control in the cortico-basal ganglia-thalamocortical circuit: complex modulation and its interplay with working memory and decision-making. Neuron.

[16] Walker CK, Roche JK, Sinha V, Roberts RC (2018). Substantia nigra ultrastructural pathology in schizophrenia. Schizophr Res.

[17] Maia TV, Frank MJ (2017). An integrative perspective on the role of dopamine in schizophrenia. Biol Psychiatry.

[18] Oldfield RC (1971). The assessment and analysis of handedness: the edinburgh inventory. Neuropsychologia.

[19] Sass H., Wittchen H.U., Zaudig M., I. H (2003) Diagnostisches und statistisches manual psychischer störungen dsm-iv-tr: Textrevision. Hogrefe Verlag; Auflage: 1 (1. Januar 2003)

[20] Hirjak D, Kubera KM, Northoff G, Fritze S, Bertolino AL, Topor CE, Schmitgen MM, Wolf RC (2019). Cortical contributions to distinct symptom dimensions of catatonia. Schizophr Bull.

[21] Hirjak D, Rashidi M, Kubera KM, Northoff G, Fritze S, Schmitgen MM, Sambataro F, Calhoun VD, Wolf RC (2020). Multimodal magnetic resonance imaging data fusion reveals distinct patterns of abnormal brain structure and function in catatonia. Schizophr Bull.

[22] Kay SR (1990). Positive-negative symptom assessment in schizophrenia: psychometric issues and scale comparison. Psychiatr Q.

[23] Overall JE, Gorham DR (1962). The brief psychiatric rating scale (bprs). Psychol Rep.

[24] Guy W (1976) Clinical global impressions, ECDEU assessment manual for psychopharmacology, revised (DHEW Publ. No. ADM 76-338). National Institute of Mental Health, Rockville, pp 218–222

[25] DSM-III.R. DKuDddusMpSr (1989). Gaf-skala: global assessment of functioning scale.

[26] Simpson GM, Angus JW (1970). A rating scale for extrapyramidal side effects. Acta Psychiatr Scand Suppl.

[27] Hirjak D, Thomann PA, Northoff G, Kubera KM, Wolf RC (2017). german version of the northoff catatonia rating scale (ncrs-dv): a validated instrument for measuring catatonic symptoms. Nervenarzt.

[28] Northoff G, Koch A, Wenke J, Eckert J, Boker H, Pflug B, Bogerts B (1999). Catatonia as a psychomotor syndrome: a rating scale and extrapyramidal motor symptoms. Mov Disord Off J Mov Disord Soc.

[29] Leucht S, Samara M, Heres S, Patel MX, Furukawa T, Cipriani A, Geddes J, Davis JM (2015). Dose equivalents for second-generation antipsychotic drugs: the classical mean dose method. Schizophr Bull.

[30] Cuesta MJ, Sanchez-Torres AM, de Jalon EG, Campos MS, Ibanez B, Moreno-Izco L, Peralta V (2014). Spontaneous parkinsonism is associated with cognitive impairment in antipsychotic-naive patients with first-episode psychosis: a 6-month follow-up study. Schizophr Bull.

[31] Molina V, Lubeiro A, Blanco J, Blanco JA, Rodriguez M, Rodriguez-Campos A, de Luis-Garcia R (2018). Parkinsonism is associated to fronto-caudate disconnectivity and cognition in schizophrenia. Psychiatry Res Neuroimaging.

[32] Dazzan P, Morgan KD, Orr KG, Hutchinson G, Chitnis X, Suckling J, Fearon P, Salvo J, McGuire PK, Mallett RM, Jones PB, Leff J, Murray RM (2004). The structural brain correlates of neurological soft signs in aesop first-episode psychoses study. Brain.

[33] Khan AR, Wang L, Beg MF (2008). Freesurfer-initiated fully-automated subcortical brain segmentation in mri using large deformation diffeomorphic metric mapping. Neuroimage.

[34] Fischl B, Dale AM (2000). Measuring the thickness of the human cerebral cortex from magnetic resonance images. Proc Natl Acad Sci USA.

[35] Fischl B, Sereno MI, Dale AM (1999). Cortical surface-based analysis. Ii: inflation, flattening, and a surface-based coordinate system. Neuroimage.

[36] Dale AM, Fischl B, Sereno MI (1999). Cortical surface-based analysis. I. Segmentation and surface reconstruction. Neuroimage.

[37] Buckner RL, Head D, Parker J, Fotenos AF, Marcus D, Morris JC, Snyder AZ (2004). A unified approach for morphometric and functional data analysis in young, old, and demented adults using automated atlas-based head size normalization: reliability and validation against manual measurement of total intracranial volume. Neuroimage.

[38] Benjamini Y, Hochberg Y (1995) Controlling the false discovery rate: A practical and powerful approach to multiple testing. J Royal Stat Soc Series B (Methodological), 57(1), 289–300. Retrieved from http://www.jstor.org/stable/2346101

[39] Paul MS, Das JS (2020). Neuroanatomy, superior and inferior olivary nucleus (superior and inferior olivary complex).

[40] Schrag A, Kingsley D, Phatouros C, Mathias CJ, Lees AJ, Daniel SE, Quinn NP (1998). Clinical usefulness of magnetic resonance imaging in multiple system atrophy. J Neurol Neurosurg Psychiatry.

[41] Suzuki M, Nakamura T, Hirayama M, Ueda M, Imai E, Harada Y, Katsuno M (2020). Relationship between cardiac parasympathetic dysfunction and the anteroposterior diameter of the medulla oblongata in multiple system atrophy. Clin Auton Res.

[42] Fritze S, Thieme CE, Kubera KM, Northoff G, Schmitgen MM, Wolf RC, Hirjak D (2020). Brainstem alterations contribute to catatonia in schizophrenia spectrum disorders. Schizophr Res.

[43] Elvsashagen T, Bahrami S, van der Meer D, Agartz I, Alnaes D, Barch DM, Baur-Streubel R, Bertolino A, Beyer MK, Blasi G, Borgwardt S, Boye B, Buitelaar J, Boen E, Celius EG, Cervenka S, Conzelmann A, Coynel D, Di Carlo P, Djurovic S, Eisenacher S, Espeseth T, Fatouros-Bergman H, Flyckt L, Franke B, Frei O, Gelao B, Harbo HF, Hartman CA, Haberg A, Heslenfeld D, Hoekstra PJ, Hogestol EA, Jonassen R, Jonsson EG, Kirsch P, Kloszewska I, Lagerberg TV, Landro NI, Le Hellard S, Lesch KP, Maglanoc LA, Malt UF, Mecocci P, Melle I, Meyer-Lindenberg A, Moberget T, Nordvik JE, Nyberg L, Connell KSO, Oosterlaan J, Papalino M, Papassotiropoulos A, Pauli P, Pergola G, Persson K, de Quervain D, Reif A, Rokicki J, van Rooij D, Shadrin AA, Schmidt A, Schwarz E, Selbaek G, Soininen H, Sowa P, Steen VM, Tsolaki M, Vellas B, Wang L, Westman E, Ziegler GC, Zink M, Andreassen OA, Westlye LT, Kaufmann T, Karolinska Schizophrenia Project c (2020). The genetic architecture of human brainstem structures and their involvement in common brain disorders. Nat Commun.

[44] McCutcheon RA, Jauhar S, Pepper F, Nour MM, Rogdaki M, Veronese M, Turkheimer FE, Egerton A, McGuire P, Mehta MM, Howes OD (2020). The topography of striatal dopamine and symptoms in psychosis: An integrative positron emission tomography and magnetic resonance imaging study. Biol Psychiatry Cogn Neurosci Neuroimaging.

[45] Ghandili M, Munakomi S (2020). Neuroanatomy, putamen.

[46] Glenthoj A, Glenthoj BY, Mackeprang T, Pagsberg AK, Hemmingsen RP, Jernigan TL, Baare WF (2007). Basal ganglia volumes in drug-naive first-episode schizophrenia patients before and after short-term treatment with either a typical or an atypical antipsychotic drug. Psychiatry Res.

[47] Gur RE, Maany V, Mozley PD, Swanson C, Bilker W, Gur RC (1998). Subcortical mri volumes in neuroleptic-naive and treated patients with schizophrenia. Am J Psychiatry.

[48] Levitt JJ, Rosow LK, Nestor PG, Pelavin PE, Swisher TM, McCarley RW, Shenton ME (2013). A volumetric mri study of limbic, associative and sensorimotor striatal subregions in schizophrenia. Schizophr Res.

[49] Andersen HG, Raghava JM, Svarer C, Wulff S, Johansen LB, Antonsen PK, Nielsen MO, Rostrup E, Vernon AC, Jensen LT, Pinborg LH, Glenthoj BY, Ebdrup BH (2020). Striatal volume increase after six weeks of selective dopamine d2/3 receptor blockade in first-episode, antipsychotic-naive schizophrenia patients. Front Neurosci.

[50] Luo Q, Chen Q, Wang W, Desrivieres S, Quinlan EB, Jia T, Macare C, Robert GH, Cui J, Guedj M, Palaniyappan L, Kherif F, Banaschewski T, Bokde ALW, Buchel C, Flor H, Frouin V, Garavan H, Gowland P, Heinz A, Ittermann B, Martinot JL, Artiges E, Paillere-Martinot ML, Nees F, Orfanos DP, Poustka L, Frohner JH, Smolka MN, Walter H, Whelan R, Callicott JH, Mattay VS, Pausova Z, Dartigues JF, Tzourio C, Crivello F, Berman KF, Li F, Paus T, Weinberger DR, Murray RM, Schumann G, Feng J, consortium I,  (2019). Association of a schizophrenia-risk nonsynonymous variant with putamen volume in adolescents: a voxelwise and genome-wide association study. JAMA Psychiat.

[51] Huang AS, Rogers BP, Sheffield JM, Jalbrzikowski ME, Anticevic A, Blackford JU, Heckers S, Woodward ND (2020). Thalamic nuclei volumes in psychotic disorders and in youths with psychosis spectrum symptoms. Am J Psychiatry.

[52] Grama S, Willcocks I, Hubert JJ, Pardinas AF, Legge SE, Bracher-Smith M, Menzies GE, Hall LS, Pocklington AJ, Anney RJL, Bray NJ, Escott-Price V, Caseras X (2020). Polygenic risk for schizophrenia and subcortical brain anatomy in the uk biobank cohort. Transl Psychiatry.

[53] Youssef HA, Waddington JL (1988). Primitive (developmental) reflexes and diffuse cerebral dysfunction in schizophrenia and bipolar affective disorder: overrepresentation in patients with tardive dyskinesia. Biol Psychiatry.

[54] Schott JM, Rossor MN (2003). The grasp and other primitive reflexes. J Neurol Neurosurg Psychiatry.

[55] Walterfang M, Velakoulis D (2005). Cortical release signs in psychiatry. Aust NZ J Psychiatry.

[56] Paulson GW (1968). An evaluation of the permanence of the "tardive dyskinesias". Dis Nerv Syst.

[57] Paulson G, Gottlieb G (1968). Development reflexes: the reappearance of fetal and neonatal reflexes in aged patients. Brain.

[58] Moustafa AA, McMullan RD, Rostron B, Hewedi DH, Haladjian HH (2017). The thalamus as a relay station and gatekeeper: relevance to brain disorders. Rev Neurosci.

[59] Praharaj SK, Arora M, Gandotra S (2006). Clozapine-induced sialorrhea: pathophysiology and management strategies. Psychopharmacology.

[60] Hirjak D, Meyer-Lindenberg A, Kubera KM, Thomann PA, Wolf RC (2018). Motor dysfunction as research domain in the period preceding manifest schizophrenia: a systematic review. Neurosci Biobehav Rev.

[61] Peralta V, Cuesta MJ (2011). Neuromotor abnormalities in neuroleptic-naive psychotic patients: antecedents, clinical correlates, and prediction of treatment response. Compr Psychiatry.

[62] Smeland OB, Shadrin A, Bahrami S, Broce I, Tesli M, Frei O, Wirgenes KV, O'Connell KS, Krull F, Bettella F, Steen NE, Sugrue L, Wang Y, Svenningsson P, Sharma M, Pihlstrom L, Toft M, O'Donovan M, Djurovic S, Desikan R, Dale AM, Andreassen OA (2021). Genome-wide association analysis of parkinson's disease and schizophrenia reveals shared genetic architecture and identifies novel risk loci. Biol Psychiatry.

[63] Kirouac GJ (2015). Placing the paraventricular nucleus of the thalamus within the brain circuits that control behavior. Neurosci Biobehav Rev.

[64] Armstrong DM (1986). Supraspinal contributions to the initiation and control of locomotion in the cat. Prog Neurobiol.

